# Improving survival in recurrent medulloblastoma: earlier detection, better treatment or still an impasse?

**DOI:** 10.1038/bjc.1998.220

**Published:** 1998-04

**Authors:** E. Bouffet, F. Doz, M. C. Demaille, P. Tron, H. Roche, D. Plantaz, A. Thyss, J. L. Stephan, O. Lejars, E. Sariban, M. Buclon, J. M. ZÃ¼cker, M. Brunat-Mentigny, J. L. Bernard, J. C. Gentet

**Affiliations:** Department of Paediatric Oncology, Centre Leon BÃ©rard, Lyon, France.

## Abstract

Early detection of relapse has been advocated to improve survival in children with recurrent medulloblastoma. However, the prognostic factors and the longer term outcome of these patients remains unclear. Pattern of recurrences were analysed in three consecutive protocols of the SociÃ©tÃ© FranÃ§aise d'Oncologie PÃ©diatrique (1985-91). A uniform surveillance programme including repeated lumbar puncture combined with computerized tomography (CT) or magnetic resonance imaging (MRI) scan was applied for all registered patients. Forty-six out of 116 patients had progressive or recurrent disease. The median time from diagnosis to recurrence was 10.5 months and 76% relapses occurred during the first 2 years. Seventeen patients had asymptomatic relapses that were detected by the surveillance protocol. Forty-one patients were treated at time of progression. Twenty-three responded to salvage therapy and 11 achieved a second complete remission. The median survival time after progression was 5 months (<1-41 months), and only two patients remained alive at time of follow-up. Length of survival is primarily related to some specific patterns of relapse (time from diagnosis to recurrence, circumstances of relapse, extent of relapse) and to the response to salvage therapy. No evidence of long-term benefit appeared from any form of treatment.


British Journal of Cancer (1998) 77(8), 1321–1326
© 1998 Cancer Research Campaign

### Improving survival in recurrent medulloblastoma: earlier detection, better treatment or still an impasse?

E Bouffet[1], F Doz[2], MC Demaille[3], P Tron[4], H Roche[5], D Plantaz[6], A Thyss[7], JL Stephan[8], O Lejars[9], E Sariban[10], M Buclon[11], JM Zücker[2], M Brunat-Mentigny[1], JL Bernard[12], and JC Gentet[12]

[1]Department of Paediatric Oncology, Centre Leon Bérard, 28, rue Laennec, 69373 Lyon Cedex 08, France; [2]Department of Paediatric Oncology, Institut Curie, Paris, France; [3]Department of Paediatric Oncology, Centre O. Lambret, Lille, France; [4]Department of Paediatrics, Hôpital Charles Nicolle, Rouen, France; [5]Department of Paediatric Oncology, Centre Claudius Regaud, Toulouse, France; [6]Department of Paediatric Oncology, Hôpital de la Tronche, Grenoble, France; [7]Department of Paediatric Oncology, Centre Antoine Lacassagne, Nice, France; [8]Department of Paediatric Oncology, Hôpital Nord, Saint Etienne, France; [9]Department of Paediatric Haematology-Oncology, Hôpital Reine Fabiola, Brussels, Belgium; [10]Department of Paediatrics, CHU of Tours, France; [11]Unité de Biostatistiques et Evaluation thérapeutique, Centre Léon Bérard, Lyon, France; [12]Department of Paediatric Oncology, Hôpital de la Timone, Marseille, France

**Summary** Early detection of relapse has been advocated to improve survival in children with recurrent medulloblastoma. However, the prognostic factors and the longer term outcome of these patients remains unclear. Pattern of recurrences were analysed in three consecutive protocols of the Société Française d'Oncologie Pédiatrique (1985–91). A uniform surveillance programme including repeated lumbar puncture combined with computerized tomography (CT) or magnetic resonance imaging (MRI) scan was applied for all registered patients. Forty-six out of 116 patients had progressive or recurrent disease. The median time from diagnosis to recurrence was 10.5 months and 76% relapses occurred during the first 2 years. Seventeen patients had asymptomatic relapses that were detected by the surveillance protocol. Forty-one patients were treated at time of progression. Twenty-three responded to salvage therapy and 11 achieved a second complete remission. The median survival time after progression was 5 months (<1–41 months), and only two patients remained alive at time of follow-up. Length of survival is primarily related to some specific patterns of relapse (time from diagnosis to recurrence, circumstances of relapse, extent of relapse) and to the response to salvage therapy. No evidence of long-term benefit appeared from any form of treatment.

**Keywords**: medulloblastoma; relapse; prognosis; salvage therapy

Survival rate in medulloblastoma is now around 60% at 10 years (Evans et al, 1990; Bailey et al, 1995; Gentet et al, 1995). Thus, 40% of children will develop recurrent disease despite surgery, chemotherapy and radiotherapy. The prognosis of these patients has in the past been dismal, with only anecdotal reports describing prolonged survival after relapse (Miyagami et al, 1993; Mahoney et al, 1996). It has been suggested that early identification of recurrence was associated with a longer survival (Mendel et al, 1996). Over the last years, the value of surveillance scanning in children with medulloblastoma has generated tremendous interest and controversy (Torres et al, 1994; Friedman and Kun, 1995; Steinbok et al, 1996). There are several reasons why this debate remains open. First, the potential benefit of such surveillance scanning programmes has to be balanced against its economical and psychological impact. Second, differences in survival may reflect inherent differences in tumour aggressiveness rather than the result of earlier detection. Finally, other independent factors may influence the outcome of children with recurrent medulloblastoma. The aim of this study was to determine the factors influencing the length of survival in an unselected group of children registered in three consecutive cooperative protocols of the Société Française d'Oncologie Pédiatrique (SFOP).

*Received 11 April 1997*
*Revised 1 July 1997*
*Accepted 25 September 1997*

*Correspondence to:* E Bouffet, Institute of Child Health, Bristol Royal Hospital for Sick Children, St Michael's Hill, Bristol BS2 8BJ, UK

### PATIENTS AND METHODS

One-hundred and sixteen patients aged less than 20 years were registered in the M7, M8 and M9 protocols, three consecutive protocols for medulloblastoma of the SFOP. Eleven institutions participated.

All patients underwent craniotomy with removal of as much disease as possible. The pathological diagnosis of medulloblastoma was mandatory before initiation of the protocol. Initial staging procedure included clinical examination, review of the operative notes, cranial computerized tomography (CT) or magnetic resonance imaging (MRI) scan at day 20 after surgery, and CSF examination at day 21 at time of myelogram. Patients were classified in two groups: high-risk patients with either brain stem involvement, incomplete resection or metastasis and low-risk patients with localized disease and complete resection. Thirty-three of the 116 patients had initially metastatic disease.

The M7 protocol (1985–88) (Gentet et al, 1995) included two courses of the '8 in 1' regimen (Pendergrass et al, 1987) on day 8 and day 21 after surgery. High-dose methotrexate (12 g $m^{-2}$) with subsequent folinic acid rescue was given at day 35 and 42. Four additional courses of '8 in 1' were given once monthly after radiation therapy for high-risk patients. The M8 protocol (1988–89) included three courses of '8 in 1' before radiation therapy and three additional courses of '8 in 1' after radiation therapy for high-risk

This work has been presented at the SIOP XXVII Meeting in Montevideo (October 1995).

patients. The M9 protocol (1989–91) included six courses of '8 in 1' followed by radiation therapy for both low-risk and high-risk patients. Radiation was either initiated during the chemotherapy, overlapping with the two high-dose methotrexate courses in the M7 protocol, after three courses of '8 in 1' in the M8 protocol or after six courses of '8 in 1' in the M9. Radiation was administered to the cranial field at a daily dose of 1.8 Gy with five weekly fractions up to a recommended dose of 27 Gy. A 27-Gy boost was given to the posterior fossa. Spinal irradiation was delivered with a posterior field at the same fractionation, up to 35 Gy. An additional 10-Gy boost was given locally in the case of spinal or supratentorial metastases.

All patients were followed clinically during and after treatment. CT or MRI scan of the head with and without administration of contrast material, was obtained 2 months after the completion of the radiotherapy, and then every 4 months during the first 2 years unless additional examinations were indicated clinically. The scans were then repeated at 6 monthly intervals. CSF was examined 2 months after the completion of radiotherapy, every 2 months during the first year, and every 4 months during the second year. Progression was defined as any new lesion detected in any of the examinations. A complete restaging (CSF examination, CT scan or MRI of the brain, and myelogram or MRI of the spinal axis) was performed for relapsing patients. Failures were analysed in two different ways: (a) according to the site into local, distant and combined relapses; and (b) according to the extent they were classified as 'isolated' when the staging revealed a single site of progression, and 'combined' in all other situations. Over this period, guidelines for salvage therapy were based on the current SFOP protocols, i.e. phase II studies of ifosfamide or etoposide and carboplatin as described previously (Chastagner et al, 1993; Gentet et al, 1994), and high-dose chemotherapy followed by autologous bone marrow rescue for responding patients. Sixty-eight patients were treated according to the M7 protocol, 19 according to the M8 and 29 according to the M9. Thirty-three out of the 116 patients had initially metastatic disease.

We examined the following factors for prognostic significance: age at diagnosis, sex, initial staging, site of failure, time from diagnosis to relapse, patterns of detection and response to salvage therapy. For all the variables examined, the results are reported in terms of survival. Survival was measured from the time of relapse to the date of death or last follow-up. Survival curves were drawn using the Kaplan and Meier method (Kaplan and Meier, 1958). As nearly all patients died of progression, length of survival rather than survival was considered with regard to statistical analysis. In the univariate analysis the prognostic factors were compared using the log-rank test. Multivariate analysis was performed using the Cox proportional hazard model. Statistical analyses were conducted according to the procedure of the BMDP package (BMDP Statistical Software, Los Angeles, CA, USA).

### RESULTS

### Patterns of recurrences

The median follow-up for recurrence-free patients is 102 months. Forty-six patients relapsed. The age and sex of patients with and without recurrence were similar. Considering the disease extent at the time of initial diagnosis, 23 patients had metastatic disease and 23 had localized disease. Relapses occurred within a median time of 315 days from surgery, ranging from 41 days to 81 months.

**Table 1** Total number of sites in 45 relapsing patients and distribution of sites in 22 patients with isolated site of relapse

| | Total | Isolated |
|---|---|---|
| Local | 20 | 10 |
| CSF | 20 | 4 |
| Spinal | 15 | 0 |
| Supratentorial | 19 | 8 |
| Extraneural | 3 | 0 |
| Number of occurrences | 77 | 22 |

CSF, cerebrospinal fluid.

There was no significant difference between the timing of relapse between the three protocol groups. Twenty-five patients (54%) had tumour recurrence during the first year after diagnosis, ten (22%) during the second year, three during the third year, four during the fourth year and three later on. Overall, 76% of recurrences occurred during the first 2 years. Six patients developed progressive disease during treatment. Subclinical recurrences were detected in 17 patients (37%) during surveillance procedures at a median time of 15 months from diagnosis (mean 21 months, range 5–30 months). Clinical recurrences occurred earlier, with a median timing of 9 months from diagnosis (mean 17.5 months, range 1.5–81 months), although this difference was not statistically significant ($P = 0.41$). Extensive restaging was performed in 42 out of the 46 patients, but sites of relapse were reported and analysed in 45 patients, three patients having had incomplete investigations (Table 1). Local relapse was reported in ten patients, distant relapse in 25 patients and local + distant in ten patients. Twenty-two patients had isolated relapses: ten in the primary tumour site, eight in the supratentorial area and four had isolated CSF relapse. Twenty-three patients had a combined relapse. This included posterior fossa in ten, CSF in 16, supratentorial area in 11, spinal axis in 15 and systemic metastases in three patients.

### Treatment of relapse

Treatment at time of relapse varied according to each institution, taking into account the potential for resection or reirradiation, and the wishes of the family. Treatment modalities are summarized in Table 2. Five patients received only steroids and analgesics. Seven patients had an attempt to excisional surgery for local (four patients), supratentorial (two patients) or spinal (one patient) relapse. Eight received radiotherapy. Two patients with early progression during sandwich therapy received craniospinal radiotherapy, four had a supratentorial boost (including two patients after surgical removal), one received a 15-Gy spinal reirradiation, and one patient with a local relapse had radiosurgery using 'gamma-knife'. Thirty-six patients were treated using chemotherapy. Twenty-three of them (64%) received etoposide and carboplatin as first- or second-line salvage treatment. Other regimens included etoposide and cyclophosphamide, '8 in 1', MOPP, etoposide and cisplatin, or ifosfamide. Response to chemotherapy was assessed according to the SIOP criteria (Gnekow, 1995). Eighteen patients (50% of chemotherapy-treated patients) achieved partial or complete response with chemotherapy. Nine responding patients (two complete and seven partial responders) received high-dose chemotherapy followed by bone marrow rescue. Regimens for high dose chemotherapy were either BCNU, carboplatin and melphalan (four patients), etoposide, carboplatin and melphalan (three patients) or etoposide and thiotepa (two patients).

© Cancer Research Campaign 1998

**Table 2** Treatment modalities and response to salvage therapy according to the type of recurrence (among 45 patients with reported sites)

| | Number of patients | Patients treated | Chemotherapy | Surgery | Radiotherapy | HDC | Response (%)[a] |
|---|---|---|---|---|---|---|---|
| Local | 10 | 10 | 9 | 4 | 1 | 1 | 6 (60) |
| Distant | 10 | 8 | 8 | 1 | 2 | 4 | 3 (37) |
| Local+distant | 25 | 23 | 19 | 2 | 5 | 4 | 13 (56) |
| Combined | 23 | 20 | 18 | 1 | 4 | 4 | 7 (35) |
| Isolated | 22 | 21 | 18 | 6 | 4 | 5 | 15 (71) |

HDC, high-dose chemotherapy. [a]Percentage according to the number of treated patients.

**Figure 1** Overall survival in months from time of relapse ($n$ = 46 patients)

Overall, among 41 patients treated at time of relapse, 11 (27%) patients achieved a second complete remission, eight had a second partial remission, and four had a transient response. Median progression-free survival was 11 months for responding patients. Three patients had stable disease, and 16 progressed despite treatment. One responding patient died of toxicity after high-dose chemotherapy.

### Outcome

Forty-four patients died. The median survival time from relapse is 5 months (Figure 1). Two patients remain alive, only one is disease free. Thirteen patients survived over 1 year after tumour recurrence. Among these 13 patients, ten had isolated relapses: six (out of eight) with supratentorial relapse, three (out of four) with isolated CSF relapse, and one (out of ten) with local relapse. Among the prognostic factors analysed, age, sex, initial staging and location of relapse (local, distant, local + distant) were not significant (Tables 3 and 4). The 23 patients relapsing beyond 315 days after diagnosis had a longer survival than the 23 patients with early relapses. Patients with relapses detected by surveillance scanning had a longer survival than patients with symptomatic relapse (Figure 2). Patients with isolated relapse had a significantly longer survival than patients with combined relapse (Figure 3). The median survival time for the 12 patients with either isolated CSF or isolated supratentorial failures was 17.8 months vs 4.7 months for the 34 other patients ($P$ = 0.00004). Patients with isolated relapses were more likely to respond to salvage therapy (response rate: 71% vs 35%, $P$ = 0.03). Patients responding to salvage therapy had a significantly better survival than non-responders. Survival time did not differ between carboplatin and non-carboplatin-containing regimens ($P$ = 0.42). Patients treated with high-dose chemotherapy had a median survival of 14.7 months, which did not significantly differ from patients responding to conventional therapy ($P$ = 0.78).

We analysed the effects of clinical parameters and treatment in a multivariate analysis of the five most significant factors (i.e. time to relapse, circumstances of relapse, extent of relapse, radiation therapy and response to treatment). The extent of relapse and response to salvage therapy were the only independent factors for the prediction of the length of survival.

**Table 3** Univariate and multivariate analysis of survival according to clinical variables and treatment modalities

| Variable | Univariate analysis $P$ | multivariate analysis $P$ |
|---|---|---|
| Age (<6/>6) | 0.59 | NS |
| Sex | 0.30 | NS |
| Initial staging (localized/metastatic) | 0.28 | NS |
| Time to relapse (<315 days/>315 days) | 0.004 | NS |
| Surveillance relapse/clinical relapse | 0.0008 | NS |
| Local/distant/local+distant relapse | 0.41 | NS |
| Isolated/combined relapse | 0.003 | 0.0027 |
| Treatment modality | | |
| Radiotherapy | 0.02 | NS |
| Surgery | 0.08 | NS |
| BMT | 0.19 | NS |
| Response to treatment | 0.000001 | <0.0001 |

BMT, bone marrow transplantation.

© Cancer Research Campaign 1998

**Table 4** Median survival time by subgroup

| Variable | Patient number | Median survival | Variable | Patient number | Median survival |
|---|---|---|---|---|---|
| <315 days | 23 | 10.5 | >315 days | 23 | 4.5 |
| Subclinical | 17 | 13.7 | Symptomatic | 29 | 4.3 |
| Isolated | 22 | 11.6 | Combined | 24 | 4.4 |
| RT[a] | 8 | 16.5 | No RT[a] | 33 | 5.7 |
| BMT[b] | 9 | 14.7 | Response/no BMT[b] | 14 | 10.5 |
| Responders[a] | 23 | 11.5 | NR/stable[a] | 19 | 4.3 |

[a]Only 41 patients treated; [b]among 23 responding patients. RT, radiotherapy; BMT, bone marrow transplantation. NR, non-responders.

**Figure 2** Survival according to circumstances of relapse (—) symptomatic, 29 patients; (- - -) surveillance relapse, 17 patients

**Figure 3** Survival according to the extent of the disease at time of relapse (—) single site of relapse, 22 patients; (- - -) multiple sites, 23 patients

### DISCUSSION

Several studies have analysed the patterns of relapse in medulloblastoma (Silverman and Simpson, 1982; Wara et al, 1994). However, little is known about the outcome and the factors influencing survival of relapsing patients, and most published data relate to selected cases or pilot studies of salvage therapies. Our study analyses an unselected population from time of recurrence. Its weakness is to analyse retrospectively patients treated in a heterogenous way. However, no patient with recurrent tumour was excluded from analysis. The 6 years follow-up from last registration allows some conclusions to be drawn.

The median time from initial diagnosis to progression does not differ from previous reports and approaches 1 year (Evans et al, 1990; Wara et al, 1994; Bailey et al, 1995; Gentet et al, 1995). Most of the relapses (76%) occurred during the first 2 years after diagnosis. In the present study, the overall 7-year progression-free survival in these three consecutive protocols is 60%. This means that, in our experience, the 5-year survival of patients who are alive recurrence-free at 2 years is 90%. Three relapses occurred after 4 years. Similar late recurrences have been reported previously and account for 2–10% of treatment failures in medulloblastoma (Latchaw et al, 1985; Lefkowitz et al, 1988). Regardless of the circumstances of detection, the disease-free time correlated positively with survival time after recurrence. Such a result is in accordance with other paediatric and adult malignancies (Elson et al, 1988; Grundy et al, 1989; Leivonen and Kalima, 1991).

In our study, 17 (37%) of the patients had subclinical relapses detected on surveillance imaging or by lumbar puncture performed during the follow-up. This incidence ranges between Torres et al (1994) (17%) and Mendel et al (1996) (76%). This might be due to some differences in the timing of surveillance scanning, and the use of different procedures of surveillance such as CT scan, MRI scan or repeated lumbar punctures. There has been some controversy about the role of surveillance scanning and guidelines for surveillance remain controversial in medulloblastoma. A gain in survival in patients with subclinical detection has been reported by some authors (Friedman and Kun, 1995; Mendel et al, 1996). However, other authors argue that the outcome is uniformly poor regardless of the circumstances of detection (Torres et al, 1994; Steinbok et al, 1996). Such differences in clinical presentation have also been described in newly diagnosed medulloblastoma patients, larger tumours and seeding being associated with a shorter onset of symptoms (Halperin and Friedman, 1996). This suggests that the biology of the tumour might influence the clinical presentation of relapse and its detection. In our experience, univariate analysis points out a longer survival time for patients with subclinical relapse. However, the multivariate analysis weakens this significance and highlights both the importance of the type of relapse and the response to salvage treatment. It is still not certain that surveillance scanning may benefit patients with medulloblastoma and for the same reasons the role of follow-up CSF sampling remains debatable.

The pattern of relapse influences the outcome. Classically, relapses are described as local, distant and local + distant relapses. No difference in outcome is observed in this study using this classification. Our proposal to divide failures into isolated vs combined relapses provides additional information. This is particularly clear in patients with isolated CSF and supratentorial

© Cancer Research Campaign 1998

relapses. The former all had subclinical recurrences possibly related to biological features of slowly growing tumours. The latter had relapses that might be amenable to chemotherapy, surgery or additional radiation therapy. This subgroup of patients has a longer survival time and is likely to respond to salvage therapy.

Medulloblastoma has proven to be chemosensitive (Finlay and Goins, 1987). However, despite a high response rate, responses at time of relapse are often transient. This has led to the assessment of high-dose therapy for responding patients using various regimens (Kalifa et al, 1992; Finlay et al, 1996; Mahoney et al, 1996). The most promising results concern high-dose chemotherapy in relapsing infants previously non-irradiated (Dupuis-Girod et al, 1996). Encouraging preliminary survival data have been reported by Finlay (1996) with the use of high-dose chemotherapy in patients with recurrent medulloblastoma. In this experience, children with minimal tumour burden are the ones who benefit from myeloablative strategies. The present series does not support the use of high-dose chemotherapy in the small number of patients treated by this modality. However, this study was not designed to address this question. Survival time, although there may be a trend, does not significantly differ between patients treated with conventional therapy and high-dose chemotherapy. Moreover, in this series, candidates for high-dose chemotherapy have been selected among responding patients. High-dose chemotherapy still remains an experimental procedure that has yet to be shown to have any benefit over conventional chemotherapy in relapsing patients. Scheduling that exposes tumour to more sustained levels of chemotherapy might be of benefit (Ashley et al, 1996).

Median survival time in relapsing medulloblastoma is short, and only 25–30% of the patients survive more than 1 year. There may be hope with new approaches such as targeted therapy (Kemshead et al, 1992), or gene therapy (Raffel and Culver, 1994) or sequential high-dose chemotherapy. Our findings are in agreement with Torres et al (1994), who did not find any benefit in intensive surveillance scanning. Patterns of relapse strongly influence the length of survival, and the role of intensive salvage therapy in prolonging survival remains debatable. This questions the urgency to treat patients with recurrent medulloblastoma in cooperative protocols (rather than pilot studies for selected patients) in order to clarify those patients who may benefit from aggressive salvage strategies and those for whom decent palliative care is a more valuable alternative.

### ACKNOWLEDGEMENT

We are indebted to Professor Ross Pinkerton for assistance in the preparation of the manuscript.

### REFERENCES

Ashley DM, Meier L, Kerby T, Zalduondo FM, Friedman HS, Gajjar A, Kun L, Duffner PK, Smith S and Longee D (1996) Response of recurrent medulloblastoma to low-dose oral etoposide. *J Clin Oncol* **14**: 1922–1927

Bailey CC, Gnekow A, Wellek S, Jones M, Round C, Brown J, Phillips A and Neidhardt MK (1995) Prospective randomised trial of chemotherapy given before radiotherapy in childhood medulloblastoma. International Society of Paediatric Oncology (SIOP) and the (German) Society of Paediatric Oncology (GPO): SIOP II. *Med Ped Oncol* **25**: 166–178

Chastagner P, Sommelet Olive D, Kalifa C, Brunat Mentigny M, Zucker JM, Demeocq F, Baranzelli MC, Tron P, Bergeron C, Pein F and de Lumley L (1993) Phase II study of ifosfamide in childhood brain tumors: A report by the French Society of Pediatric Oncology (SFOP). *Med Ped Oncol* **21**: 49–53

Dupuis-Girod S, Hartmann O, Benhamou E, Doz F, Mechinaud F, Bouffet E, Coze C and Kalifa C (1996) Will high dose chemotherapy followed by autologous bone marrow transplantation supplant cranio-spinal irradiation in young children treated for medulloblastoma? *J Neurooncol* **27**: 87–98

Elson PJ, Witte RS and Trump DL (1988) Prognostic factors for survival in patients with recurrent or metastatic renal cell carcinoma. *Cancer Res* **48**: 7310–7313

Evans AE, Jenkin RD, Sposto R, Ortega JA, Wilson CB, Wara W, Ertel IJ, Kramer S, Chang CH and Leikin SL (1990) The treatment of medulloblastoma. Results of a prospective randomized trial of radiation therapy with and without CCNU, vincristine, and prednisone. *J Neurosurg* **72**: 572–582

Finlay JL and Goins SC (1987) Brain tumors in children. III. Advances in chemotherapy. *Am J Pediatr Hematol Oncol* **9**: 264–271

Finlay JL, Goldman S, Wong MC, Cairo M, Garvin J, Augus C, Cohen BH, Stanley P, Zimmerman RA, Bostrom B, Geyer JR, Harris RE, Sanders J, Yates AJ, Boyett JM and Packer RJ (1996) Pilot study of high-dose thiotepa and etoposide with autologous bone marrow rescue in children and young adults with recurrent CNS tumors. *J Clin Oncol* **14**: 2495–2525

Friedman HS and Kun LE (1995) More on surveillance of children with medulloblastoma. *N Engl J Med* **332**: 191

Gentet JC, Doz F, Bouffet E, Plantaz D, Roche H, Tron P, Kalifa C, Mazingue F, Sariban E, Chastagner P, Bernard JL, Brunat-Mentigny M, Raybaud C and Zucker JM (1994) Carboplatin and VP16 in medulloblastoma: a phase II study of the French Society of Pediatric Oncology. *Med Ped Oncol* **23**: 422–427

Gentet JC, Bouffet E, Doz F, Tron P, Roche H, Thyss A, Plantaz D, Stephan JL, Mottolese C, Ponvert D, Philip T, Brunat-Mentigny M, Zucker JM and Bernard JL (1995) Preirradiation chemotherapy including 'eight drugs in 1 day' regimen and high-dose methotrexate in childhood medulloblastoma: Results of the M7 French Cooperative Study. *J Neurosurg* **82**: 608–614

Gnekow AK (1995) Recommendation of the brain tumour subcommittee for the reporting of trials. *Med Ped Oncol* **24**: 104–108

Grundy P, Breslow N, Green DM, Sharples K, Evans A and D'Angio GJ (1989) Prognostic factors for children with recurrent Wilms' tumor: Results from the second and third national Wilms' tumor study. *J Clin Oncol* **7**: 638–647

Halperin EC and Friedman HS (1996) Is there a correlation between duration of presenting symptoms and stage of medulloblastoma at the time of diagnosis? *Cancer* **78**: 874–880

Kalifa C, Hartmann O, Demeocq F, Vassal G, Couanet D, Terrier Lacombwe MJ, Valteau D, Brugieres and Lemerle J and Pizer BL (1992) High-dose busulfan and thiotepa with autologous bone marrow transplantation in childhood malignant brain tumors: a phase II study. *Bone Marrow Trans* **9**: 227–233

Kaplan E and Meier P (1958) Nonparametric estimation from incomplete observation. *J Am Stat Assoc* **53**: 457–481

Kemshead JT, Papanastassiou V, Coakham HB and Pizer BL (1992) Monoclonal antibodies in the treatment of central nervous system malignancies. *Eur J Cancer* **28**: 511–513

Latchaw JP, Hahn JF, Moylan DJ, Humphries R and Meaky Jr J (1985) Medulloblastoma. Period of risk reviewed. *Cancer* **55**: 186–189

Lefkowitz IB, Packer RJ and Ryan SG (1988) Late recurrence of primitive neuroectodermal tumor/medulloblastoma. *Cancer* **62**: 826–830

Leivonen MK and Kalima TV (1991) Prognostic factors associated with survival after breast cancer recurrence. *Acta Oncol* **30**: 583–586

Mahoney DH Jr, Steuber CP, Sandbach JF, et al (1986) Extraneural metastases from medulloblastoma: long-term survival after sequentially scheduled chemotherapy and radiotherapy. *Med Ped Oncol* **14**: 329–336

Mahoney Jr DH, Strother D, Camitta B, Bowen T, Ghim T, Pick T, Wall D, Yu L, Shuster JJ and Friedman H (1996) High-dose melphalan and cyclophosphamide with autologous bone marrow rescue for recurrent/progressive malignant brain tumors in children: a pilot Pediatric Oncology Group study. *J Clin Oncol* **14**: 382–388

Mendel E, Levy ML, Raffel C, McComb JG, Pikus H, Nelson Jr MD and Ganz W (1996) Surveillance imaging in children with primitive neuroectodermal tumors. *Neurosurgery* **38**: 692–695

Miyagami M, Satoh K and Tsubokawa T (1993) A long surviving case of recurrent medulloblastoma displaying effectiveness of ACNU/vincristine chemotherapy. *J Neurooncol* **18**: 41–47

Pendergrass TW, Milstein JM, Geyer JR, Mulue AF, Kosnik EJ Morris JD, Heideman RL, Ruymann FB, Stuntz JT and Bleyer WA (1987) Eight drugs in one day chemotherapy for brain tumors: experience in 107 children and rationale for preradiation chemotherapy. *J Clin Oncol* **5**: 1221–1231

Raffel C and Culver K (1994) Gene therapy for the treatment of recurrent pediatric malignant astrocytomas with in vivo tumor transduction with the herpes simplex thymidine kinase gene/ganciclovir system. *Hum Gen Ther* **5**: 863–890

© Cancer Research Campaign 1998

Silverman CL and Simpson JR (1982) Cerebellar medulloblastoma: the importance of posterior fossa dose to survival and patterns of failure. *Int J Radiat Oncol Biol Phys* **8**: 1869–1876

Steinbok P, Hentschel S, Cochrane DD and Kestle JR (1996) Value of postoperative surveillance imaging in the management of children with some common brain tumors. *J Neurosurg* **84**: 726–732

Torres CF, Rebsamen S, Silber JH, Sutton LN, Bilaniuk LT, Zimmerman RA, Goldwein JW, Philips PC and Lange BJ (1994) Surveillance scanning of children with medulloblastoma. *N Engl J Med* **330**: 892–895

Wara WM, Le QTX, Sneed PK, Larson DA, Prados MD, Levin VA, Edwards MS and Weil MD (1994) Pattern of recurrence of medulloblastoma after low-dose craniospinal radiotherapy. *Int J Radiat Oncol Biol Phys* **30**: 551–556

 *© Cancer Research Campaign 1998*


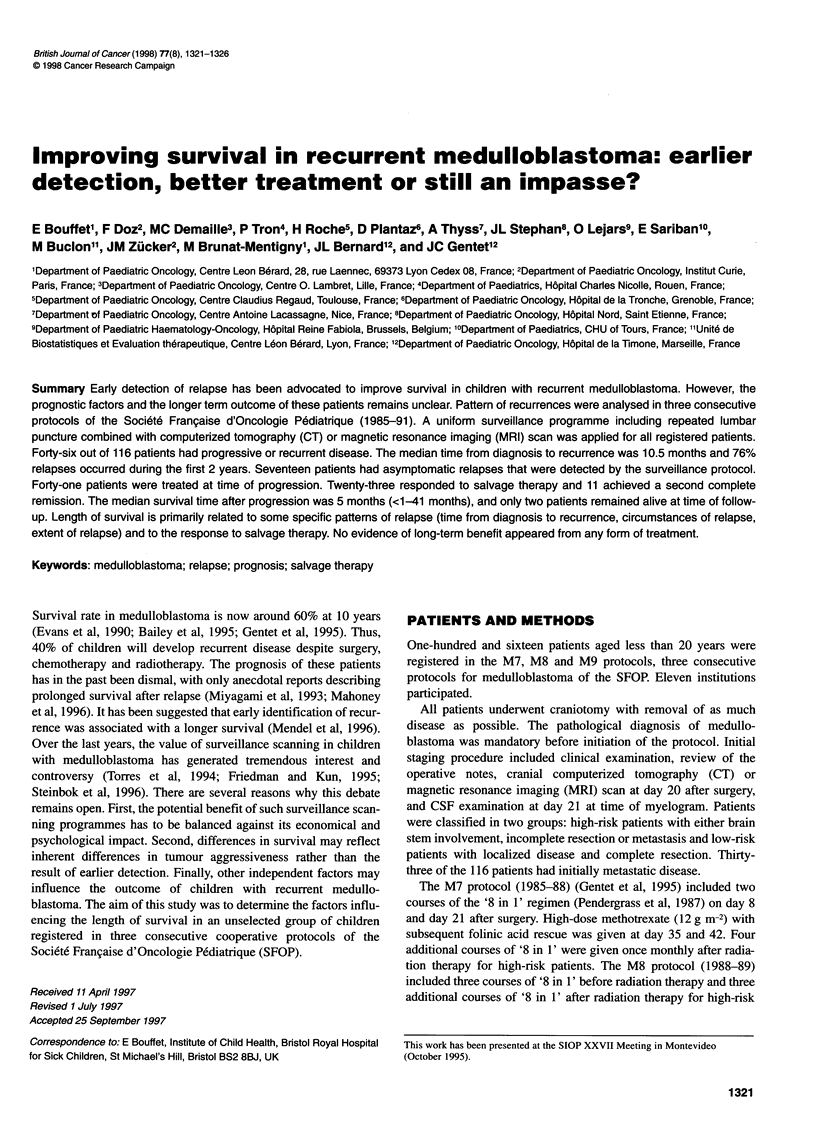

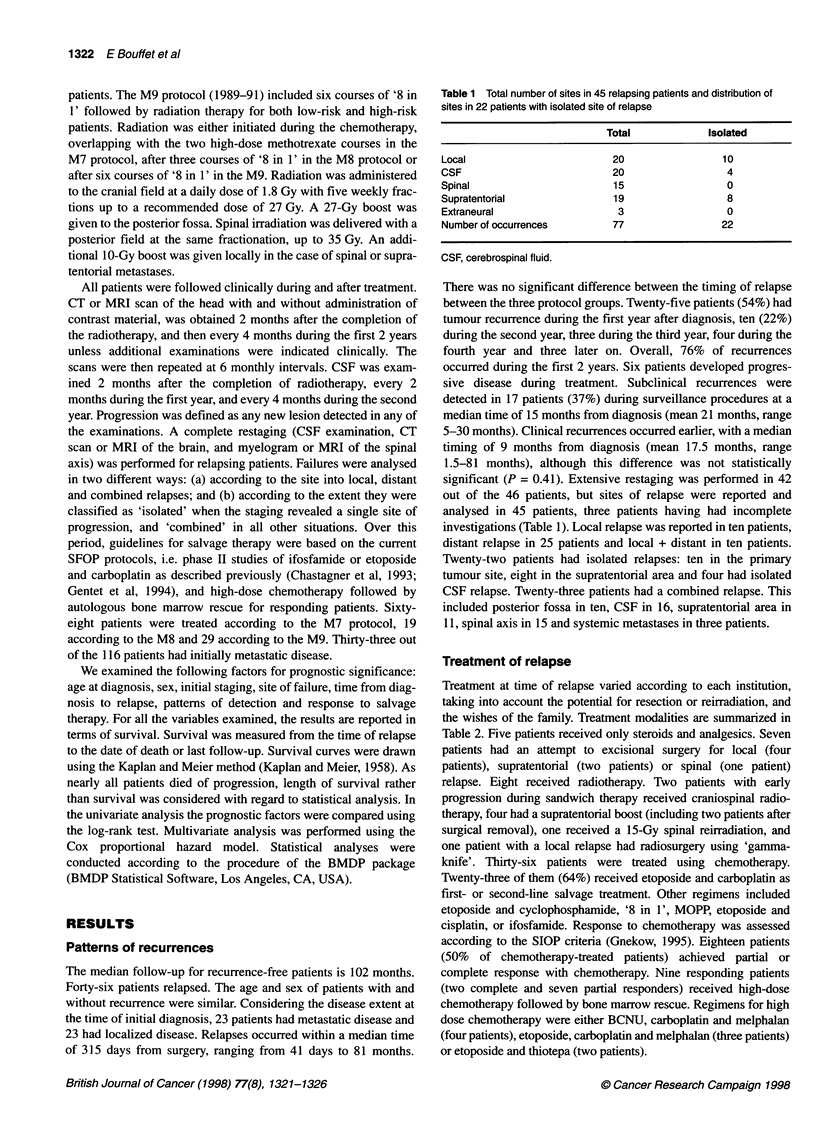

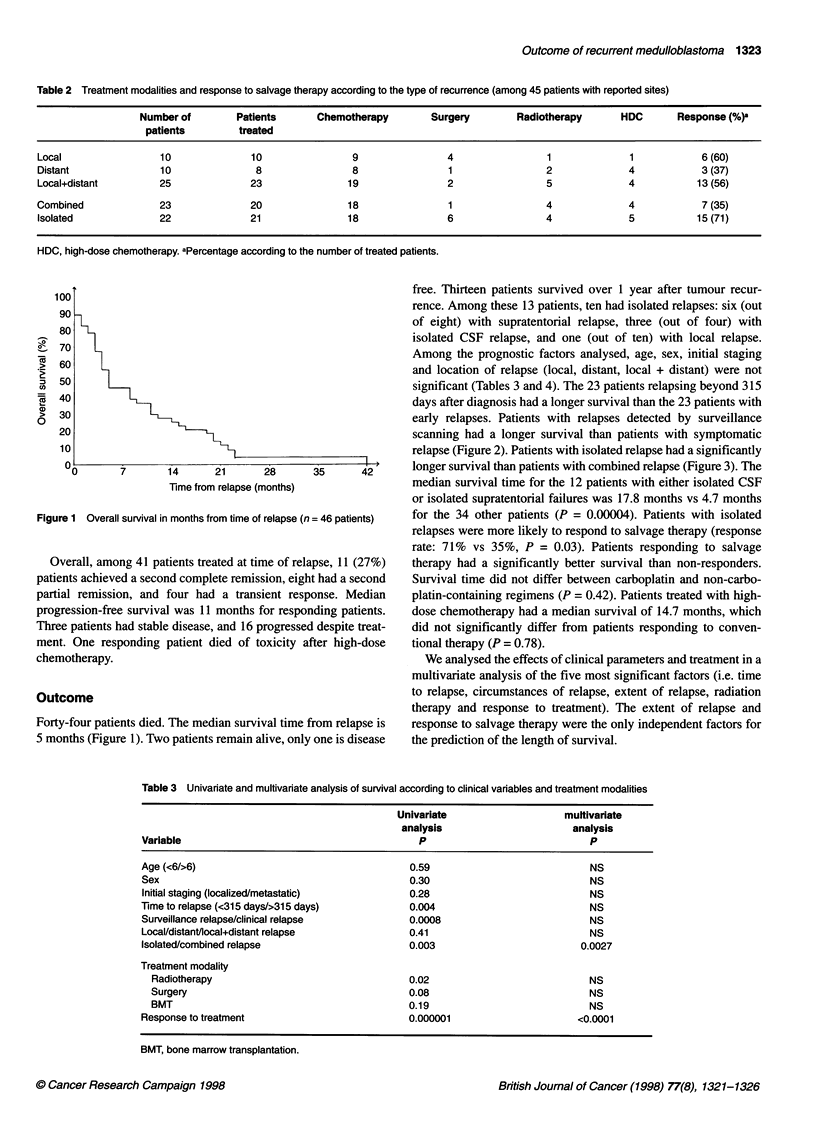

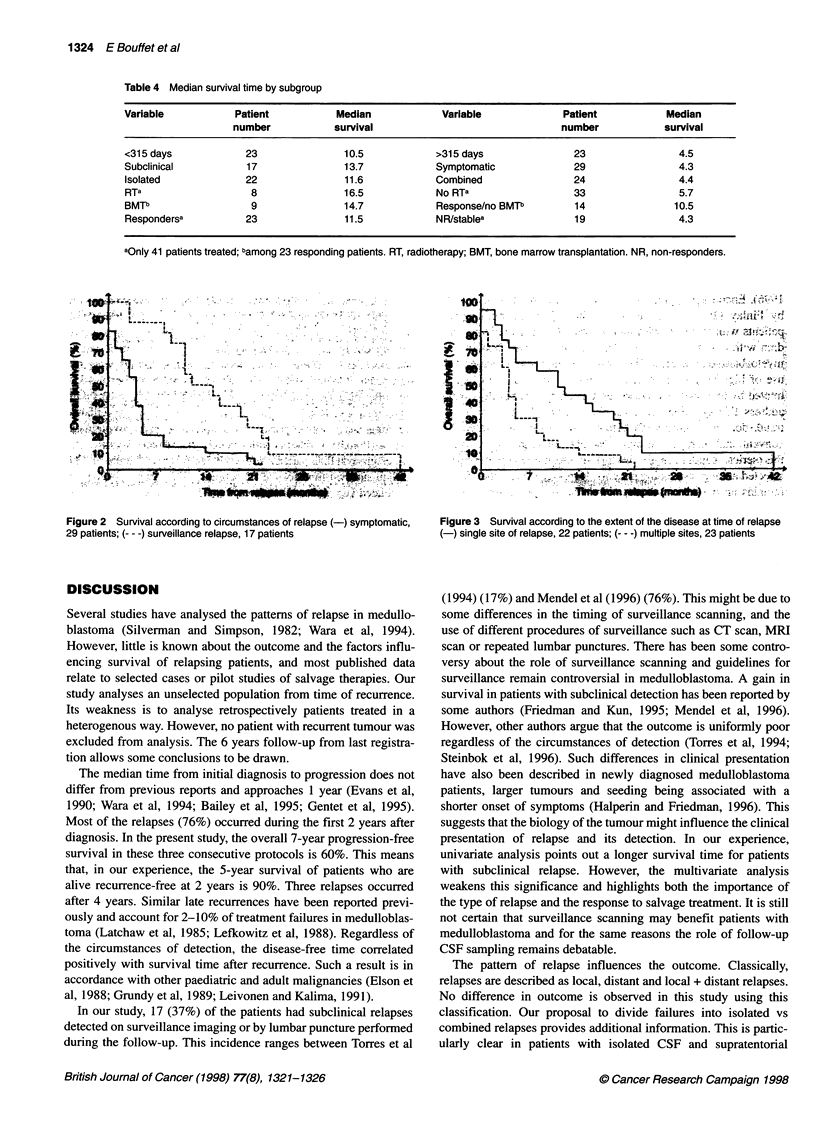

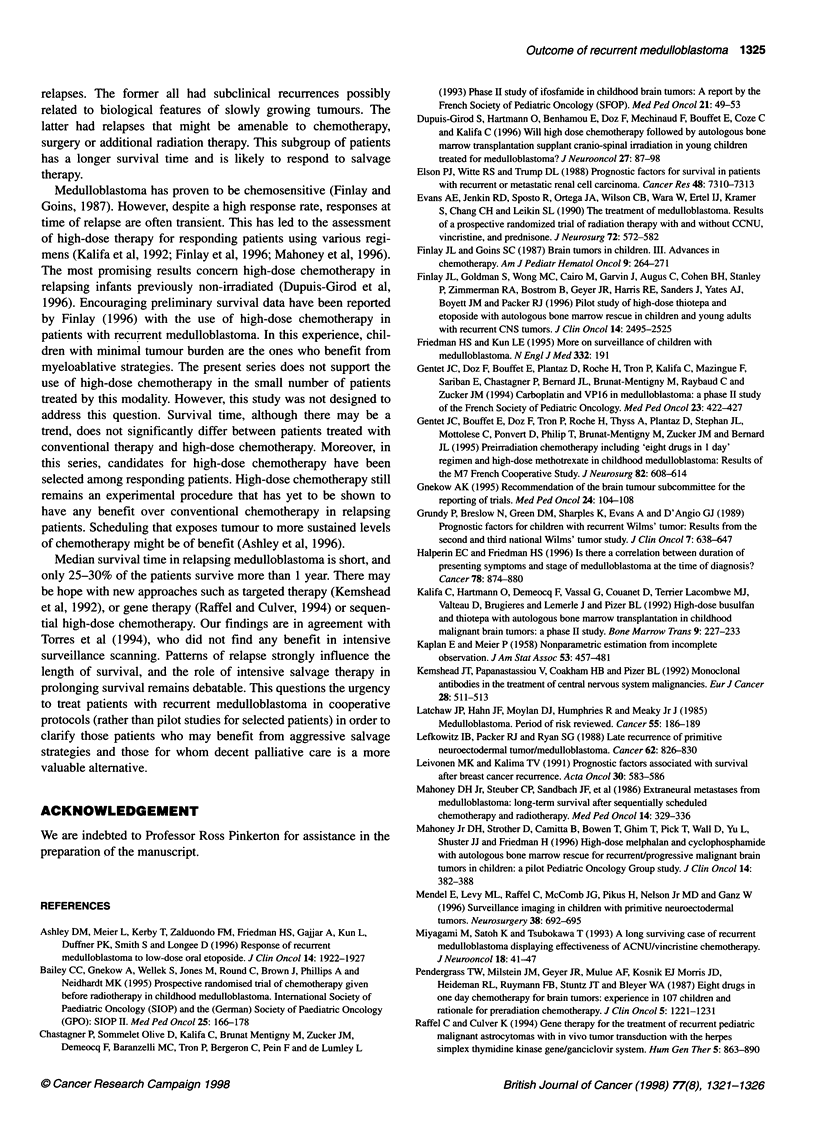

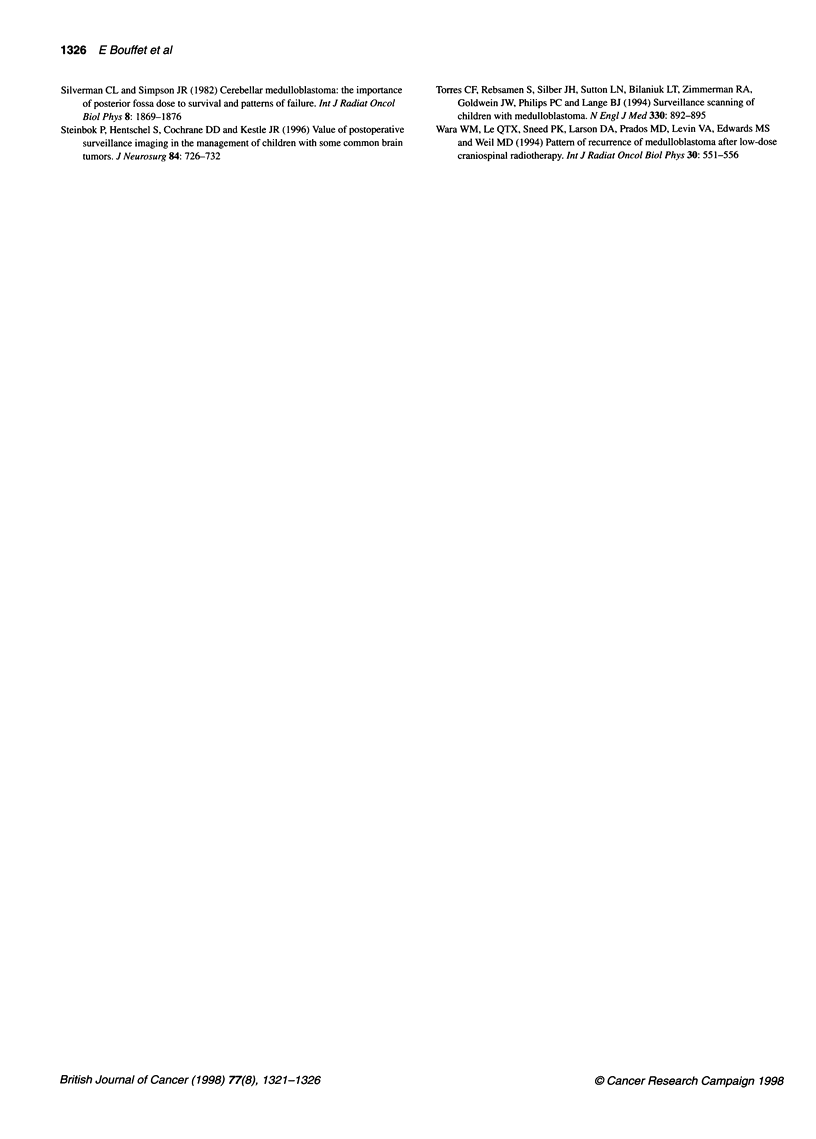


## References

[OCR_00522] Ashley D. M., Meier L., Kerby T., Zalduondo F. M., Friedman H. S., Gajjar A., Kun L., Duffner P. K., Smith S., Longee D. (1996). Response of recurrent medulloblastoma to low-dose oral etoposide.. J Clin Oncol.

[OCR_00527] Bailey C. C., Gnekow A., Wellek S., Jones M., Round C., Brown J., Phillips A., Neidhardt M. K. (1995). Prospective randomised trial of chemotherapy given before radiotherapy in childhood medulloblastoma. International Society of Paediatric Oncology (SIOP) and the (German) Society of Paediatric Oncology (GPO): SIOP II.. Med Pediatr Oncol.

[OCR_00535] Chastagner P., Sommelet-Olive D., Kalifa C., Brunat-Mentigny M., Zucker J. M., Demeocq F., Baranzelli M. C., Tron P., Bergeron C., Pein F. (1993). Phase II study of ifosfamide in childhood brain tumors: a report by the French Society of Pediatric Oncology (SFOP).. Med Pediatr Oncol.

[OCR_00542] Dupuis-Girod S., Hartmann O., Benhamou E., Doz F., Mechinaud F., Bouffet E., Coze C., Kalifa C. (1996). Will high dose chemotherapy followed by autologous bone marrow transplantation supplant cranio-spinal irradiation in young children treated for medulloblastoma?. J Neurooncol.

[OCR_00548] Elson P. J., Witte R. S., Trump D. L. (1988). Prognostic factors for survival in patients with recurrent or metastatic renal cell carcinoma.. Cancer Res.

[OCR_00551] Evans A. E., Jenkin R. D., Sposto R., Ortega J. A., Wilson C. B., Wara W., Ertel I. J., Kramer S., Chang C. H., Leikin S. L. (1990). The treatment of medulloblastoma. Results of a prospective randomized trial of radiation therapy with and without CCNU, vincristine, and prednisone.. J Neurosurg.

[OCR_00557] Finlay J. L., Goins S. C. (1987). Brain tumors in children. III. Advances in chemotherapy.. Am J Pediatr Hematol Oncol.

[OCR_00561] Finlay J. L., Goldman S., Wong M. C., Cairo M., Garvin J., August C., Cohen B. H., Stanley P., Zimmerman R. A., Bostrom B. (1996). Pilot study of high-dose thiotepa and etoposide with autologous bone marrow rescue in children and young adults with recurrent CNS tumors. The Children's Cancer Group.. J Clin Oncol.

[OCR_00569] Friedman H. S., Kun L. E. (1995). More on surveillance of children with medulloblastoma.. N Engl J Med.

[OCR_00579] Gentet J. C., Bouffet E., Doz F., Tron P., Roche H., Thyss A., Plantaz D., Stephan J. L., Mottolese C., Ponvert D. (1995). Preirradiation chemotherapy including "eight drugs in 1 day" regimen and high-dose methotrexate in childhood medulloblastoma: results of the M7 French Cooperative Study.. J Neurosurg.

[OCR_00573] Gentet J. C., Doz F., Bouffet E., Plantaz D., Roché H., Tron P., Kalifa C., Mazingue F., Sariban E., Chastagner P. (1994). Carboplatin and VP 16 in medulloblastoma: a phase II Study of the French Society of Pediatric Oncology (SFOP).. Med Pediatr Oncol.

[OCR_00587] Gnekow A. K. (1995). Recommendations of the Brain Tumor Subcommittee for the reporting of trials. SIOP Brain Tumor Subcommittee. International Society of Pediatric Oncology.. Med Pediatr Oncol.

[OCR_00591] Grundy P., Breslow N., Green D. M., Sharples K., Evans A., D'Angio G. J. (1989). Prognostic factors for children with recurrent Wilms' tumor: results from the Second and Third National Wilms' Tumor Study.. J Clin Oncol.

[OCR_00596] Halperin E. C., Friedman H. S. (1996). Is there a correlation between duration of presenting symptoms and stage of medulloblastoma at the time of diagnosis?. Cancer.

[OCR_00601] Kalifa C., Hartmann O., Demeocq F., Vassal G., Couanet D., Terrier-Lacombe M. J., Valteau D., Brugieres L., Lemerle J. (1992). High-dose busulfan and thiotepa with autologous bone marrow transplantation in childhood malignant brain tumors: a phase II study.. Bone Marrow Transplant.

[OCR_00611] Kemshead J. T., Papanastassiou V., Coakham H. B., Pizer B. L. (1992). Monoclonal antibodies in the treatment of central nervous system malignancies.. Eur J Cancer.

[OCR_00616] Latchaw J. P., Hahn J. F., Moylan D. J., Humphries R., Mealey J. (1985). Medulloblastoma. Period of risk reviewed.. Cancer.

[OCR_00620] Lefkowitz I. B., Packer R. J., Ryan S. G., Shah N., Alavi J., Rorke L. B., Sutton L. N., Schut L. (1988). Late recurrence of primitive neuroectodermal tumor/medulloblastoma.. Cancer.

[OCR_00624] Leivonen M. K., Kalima T. V. (1991). Prognostic factors associated with survival after breast cancer recurrence.. Acta Oncol.

[OCR_00628] Mahoney D. H., Steuber C. P., Sandbach J. F., Fernbach D. J. (1986). Extraneural metastases from medulloblastoma: long-term survival after sequentially scheduled chemotherapy and radiotherapy.. Med Pediatr Oncol.

[OCR_00633] Mahoney D. H., Strother D., Camitta B., Bowen T., Ghim T., Pick T., Wall D., Yu L., Shuster J. J., Friedman H. (1996). High-dose melphalan and cyclophosphamide with autologous bone marrow rescue for recurrent/progressive malignant brain tumors in children: a pilot pediatric oncology group study.. J Clin Oncol.

[OCR_00640] Mendel E., Levy M. L., Raffel C., McComb J. G., Pikus H., Nelson M. D., Ganz W. (1996). Surveillance imaging in children with primitive neuroectodermal tumors.. Neurosurgery.

[OCR_00645] Miyagami M., Satoh K., Tsubokawa T. (1994). A long surviving case of recurrent medulloblastoma displaying effectiveness of ACNU/vincristine chemotherapy.. J Neurooncol.

[OCR_00652] Pendergrass T. W., Milstein J. M., Geyer J. R., Mulne A. F., Kosnik E. J., Morris J. D., Heideman R. L., Ruymann F. B., Stuntz J. T., Bleyer W. A. (1987). Eight drugs in one day chemotherapy for brain tumors: experience in 107 children and rationale for preradiation chemotherapy.. J Clin Oncol.

[OCR_00656] Raffel C., Culver K., Kohn D., Nelson M., Siegel S., Gillis F., Link C. J., Villablanca J. G., Anderson W. F. (1994). Gene therapy for the treatment of recurrent pediatric malignant astrocytomas with in vivo tumor transduction with the herpes simplex thymidine kinase gene/ganciclovir system.. Hum Gene Ther.

[OCR_00666] Silverman C. L., Simpson J. R. (1982). Cerebellar medulloblastoma: the importance of posterior fossa dose to survival and patterns of failure.. Int J Radiat Oncol Biol Phys.

[OCR_00671] Steinbok P., Hentschel S., Cochrane D. D., Kestle J. R. (1996). Value of postoperative surveillance imaging in the management of children with some common brain tumors.. J Neurosurg.

[OCR_00676] Torres C. F., Rebsamen S., Silber J. H., Sutton L. N., Bilaniuk L. T., Zimmerman R. A., Goldwein J. W., Phillips P. C., Lange B. J. (1994). Surveillance scanning of children with medulloblastoma.. N Engl J Med.

[OCR_00681] Wara W. M., Le Q. T., Sneed P. K., Larson D. A., Prados M. D., Levin V. A., Edwards M. S., Weil M. D. (1994). Pattern of recurrence of medulloblastoma after low-dose craniospinal radiotherapy.. Int J Radiat Oncol Biol Phys.

